# Arsenic circumvents the gefitinib resistance by binding to P62 and mediating autophagic degradation of EGFR in non-small cell lung cancer

**DOI:** 10.1038/s41419-018-0998-7

**Published:** 2018-09-20

**Authors:** Jianhua Mao, Lie Ma, Yan Shen, Kongkai Zhu, Ru Zhang, Wenda Xi, Zheng Ruan, Cheng Luo, Zhu Chen, Xiaodong Xi, Saijuan Chen

**Affiliations:** 1 State Key Laboratory of Medical Genomics, Shanghai Institute of Hematology, Ruijin Hospital Affiliated to Shanghai Jiao Tong University School of Medicine, 197 Ruijin Road II, Shanghai, 200025 China; 2 Collaborative Innovation Center of Hematology, Ruijin Hospital Affiliated to Shanghai Jiao Tong University School of Medicine, 197 Ruijin Road II, Shanghai, 200025 China; 3 Shanghai Center for Systems Biomedicine, Shanghai Jiao Tong University, Shanghai, 200240 China; 4 Research Center for Experimental Medicine, Ruijin Hospital Affiliated to Shanghai Jiao Tong University School of Medicine, 197 Ruijin Road II, Shanghai, 200025 China; 5 Drug Discovery and Design Center, State Key Laboratory of Drug Research, Shanghai Institute of Materia Medica, Chinese Academy of Sciences, Shanghai, 201203 China; 6 Shanghai Institute of Hypertension, Ruijin Hospital Affiliated to Shanghai Jiao Tong University School of Medicine, Shanghai, 200025 China

## Abstract

Non-small cell lung cancer (NSCLC) is characterized by hyperexpression and/or gain-of-function mutations of the epidermal growth factor receptor (EGFR), resulting in an elevated overall kinase activity. Gefitinib is remarkably effective in patients with the L858R or ΔE746-A750-mutated of EGFR. However, drug resistance tends to develop because of the emergence of T790M mutation on EGFR. New strategies other than repressing kinase activity are thus required to treat NSCLC, thereby circumventing the resistance. In this study, arsenic trioxide (ATO) at 2 μM significantly inhibited the proliferation of the gefitinib-resistant NCI-H1975 cells of the EGFR L858R/T790M mutant compared with a modest inhibition in the gefitinib-sensitive HCC827 cells of ΔE746-A750 mutant and A549 cells of wild-type EGFR. Moreover, ATO significantly inhibited the overall kinase activity of EGFR primarily through quantitatively diminishing the EGFR in NCI-H1975 cells to an extent comparable with that reached by gefitinib in HCC827 cells. Furthermore, ATO promoted autophagic degradation of EGFR in NSCLC cells by directly binding to P62, which interacted with EGFR, preferentially the L858R/T790M mutant providing a plausible explanation for a more favorable effect of ATO on NCI-H1975 cells. Accordingly, the effect of ATO was further confirmed in the NSCLC xenograft mouse models. Our results reveal a new target for ATO with a unique molecular mechanism, i.e., ATO suppresses the overall catalytic potential of EGFR, significantly those with the L858R/T790M mutant in NCI-H1975 cells, through an autophagic degradation by interacting with P62. This study potentially offers an innovative therapeutic avenue for the NSCLC with L858R/T790M-mutated EGFR.

## Introduction

Lung cancer is a major cause of cancer death worldwide^1,2^. The elevated overall epidermal growth factor receptor (EGFR) kinase activity, as a result of the increased amount and/or the gain-of-function mutations, is largely responsible for the tumor malignancy in non-small cell lung cancer (NSCLC)^3,4^. The tyrosine kinase inhibitor (TKI) gefitinib is designed to target EGFR and has shown remarkable effects in treating NSCLC harboring EGFR with activating mutations^5,6^. Unfortunately, most cases ultimately become resistant to TKI, e.g., those who respond to gefitinib at the early stages develop resistance because of the emergence of the T790M mutation^7^. Currently, AZD9291 and EGF816 are developed to treat NSCLC harboring the L858R/T790M mutant^8,9^. However, the C797S mutant progressively becomes predominant, thereby resulting in the resistance^8,9^. Circumventing the resistance to TKI is actually the most formidable challenge in treating NSCLC. Therefore, the need for novel and effective strategies other than the EGFR kinase inhibitor is urgent.

Arsenic has gained considerable interest as a curative agent for acute promyelocytic leukemia and it is also effective in chronic myelogenous leukemia by inducing the degradation of PML-RARα^10–13^ and BCR-ABL^14–16^ through the ubiquitination-proteasome pathway. Moreover, arsenic has shown therapeutic effects on NSCLC. Clinical studies have demonstrated that the addition of arsenic trioxide (ATO) into the nebulized liquid for the treatments of lung cancer patients reduced the tumor size in ~61.9% (13/21) of the cases, with no apparent side effects^17^. Intrapleural administration of ATO in NSCLC patients with advanced large pleural effusion significantly improved the characteristics of pleural effusion ^18^. These observations suggested that ATO might contribute to the treatment of NSCLC, even though the exact effect and molecular mechanisms remain unknown.

In this study, three NSCLC cell lines were used to evaluate the effects of ATO on cell growth. Mechanisms of ATO in targeting and degrading EGFR were further explored to interpret its potential therapeutic roles.

## Results

### ATO inhibits proliferation and reduces EGFR overall tyrosine kinase activity in NSCLC cell lines

**Fig. 1:**
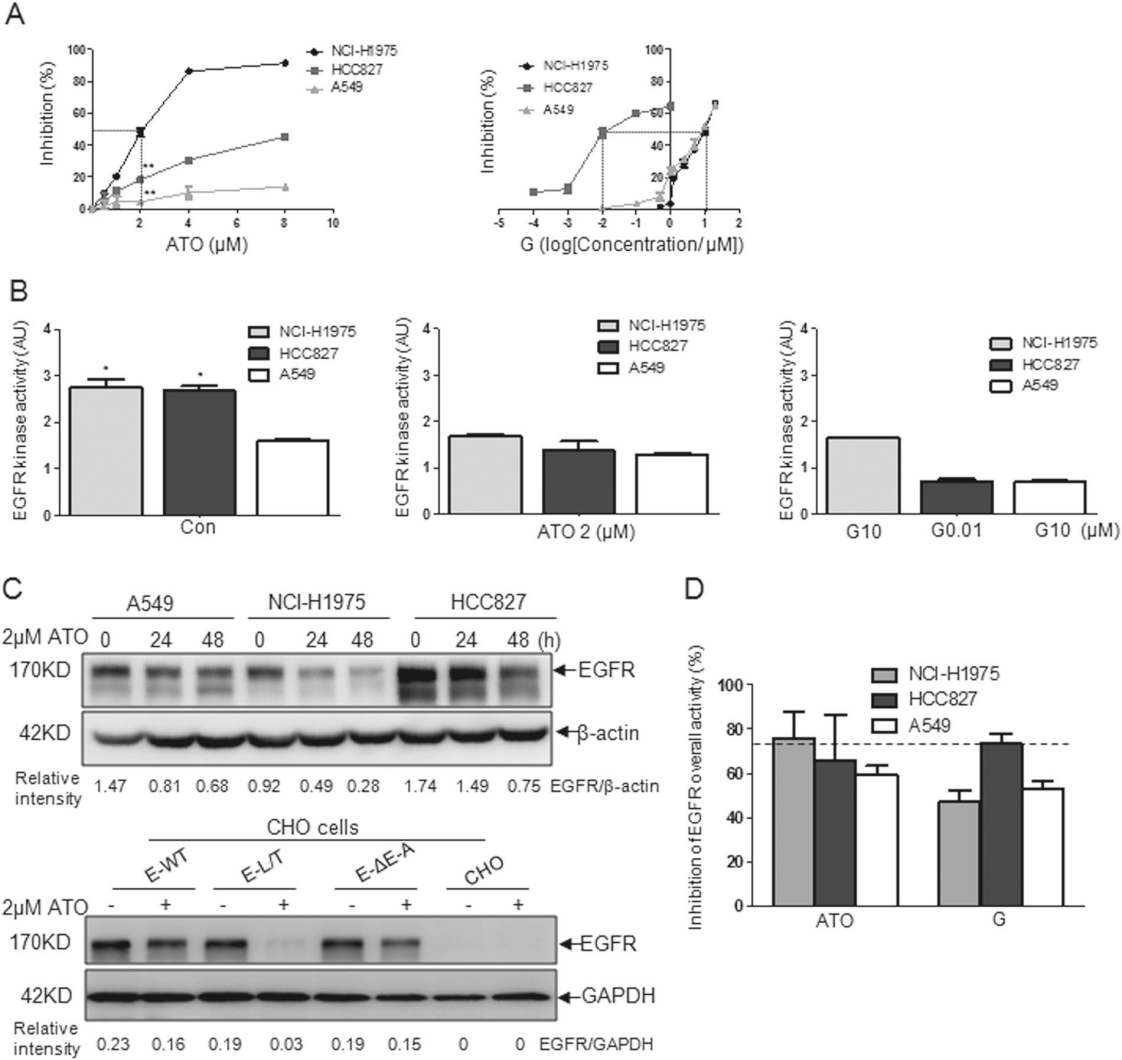
Inhibition of the proliferation and overall EGFR kinase activity by ATO in NSCLC cell lines. NCI-H1975, HCC827, and A549 cells were treated with ATO or gefitinib (g) for the indicated time. **a** Half maximal inhibitory concentrations (IC50) of arsenic and gefitinib in the three NSCLC cells were determined using CCK-8 for 48 h. The IC50 values of arsenic in NCI-H1975 were 2 µM and >8 µM in HCC827 and A549. The IC50 values of gefitinib were 0.01 µM in HCC827 and 10 µM in NCI-H1975 and A549. Black dotted line represents the IC50 concentration. **p < 0.0001 when compared with NCI-H1975 cells treated with arsenic. **b** EGFR tyrosine kinase activity was assayed when the cells were treated for 48 h using the assay kit and was calibrated based on the commercially purified EGFR WT. The OD450 (1.0) reached by 150 ng of EGFR WT was defined as one activity unit in this study. Arbitrary unit (AU) was used to represent the calibrated kinase activity (CKA), which was used to evaluate the kinase activity of the same quantity. Initial amount of EGFR for analysis in each group was 10 µg. *p < 0.05 when compared with A549 cells. **c**, **a** the NCI-H1975, HCC827, and A549 cells were treated with 2 µM of ATO at 24 and 48 h, and the expression levels of EGFR were detected by western blot analysis. **b** the CHO cells were transfected with the WT or mutant EGFR (ΔE746-A750 (ΔE-A) and L858R/T79M (L/T)), as well as the pEGFP-C1 blank vector. After 48 h of transfection, the GFP positive cells were purified by using cell sorter (BD FACSAria III). The sorted cells were seeded into the 24-well plates and treated with 2 µM of ATO for 48 h. The expression levels of EGFR were detected by western blot. The relative intensity was calculated according to the gray values of EGFR over that of β-actin or GAPDH with the Quantity One software. **d** Inhibitory rate of the overall EGFR activity was calculated using the following formula: inhibition rate = 1–EGFR overall activity after A or G treatment/EGFR overall activity before treatment. EGFR overall activity = CKA × quantity of EGFR. Original data were obtained from Fig. 1b, c and Tables S2, S3. Black dotted line indicates effective inhibitory level

Figure 1a shows that the IC50 of ATO was 2 μM for NCI-H1975 cells, compared with more than 8 μM for HCC827 and A549 cells. The IC50 value of gefitinib was 10 µM for NCI-H1975 and A549, whereas that for HCC827 cells was 0.01 µM. ATO at 2 µM and gefitinib at 0.01 µM are the conventional doses for leukemia cells and the sensitive NSCLC cells, respectively, and were thus used in the present study. Results showed that ATO and gefitinib significantly inhibited the proliferation of NCI-H1975 and HCC827 cells (Fig. 1a, S1A), respectively, confirming that NCI-H1975 is sensitive to ATO and HCC827 to gefitinib. Notably, the effect of ATO on HCC827 and A549 cells was modest. As expected, gefitinib had a subtle inhibition on NCI-H1975 cells, and A549 cells scarcely responded to gefitinib (Fig. 1a, S1A).

The EGFR tyrosine kinase activity was measured and calibrated by using the commercial wild-type (WT) EGFR protein. The initial quantity of EGFR in each group was 10 μg, and the calibrated kinase activity (CKA) was calculated (Table S1). The CKA values of NCI-H1975 and HCC827 were higher than those of A549 (Fig. 1b, *p* < 0.05), indicating the gain-of-function mutations. In the gefitinib-treated group, CKA was significantly inhibited in HCC827 cells even at a 0.01 µM (Fig. 1b). By contrast, ATO at 2 µM resulted in a similar CKA level for all the NSCLC cell lines (Fig. 1b). These data indicate that at conventional concentrations, a significant inhibitory effect on CKA only appeared in the gefitinib-treated HCC827 cells (Fig. 1b).

The quantity of EGFR is also thought to contribute to its overall kinase activity, the expression level of EGFR after arsenic treatment in NSCLC cells was thus determined. Results showed that the EGFR level after ATO treatment was downregulated in all three cell lines and most substantially in NCI-H1975 cells (Fig. 1c, Table S2). Yet the data from different NSCLC cell lines were unable to give an attribution of the differential effect of ATO to the particular EGFR mutants as the cellular backgrounds other than the EGFR mutations in the different cell lines might vary. To validate the preferential effect of ATO on the L858R/T790M mutated EGFR within a same cellular background, Chinese hamster ovary (CHO) cells, which do not express EGFR^19,20^, were transfected with the WT or mutated (ΔE746-A750 and L858R/T79M) EGFR and treated with ATO or gefitinib. The results demonstrate a similar profile of EGFR reduction (Fig. 1c) and proliferation inhibition (Fig. S1A) in response to ATO in the NSCLC cell lines and in CHO cells. These data indicate that the different EGFR proteins should be responsible for the differences in the ATO-induced reduction and thereby for the different inhibitory effect of ATO on the proliferation of the corresponding NSCLC cell lines.

Given the putative therapeutic effect of gefitinib on HCC827 cells, the inhibitory extent of the overall EGFR kinase activity in gefitinib-treated HCC827 cells was used as the criterion to evaluate the effect of ATO. The reduced extent of the overall kinase activity in ATO-treated NCI-H1975 cells met the criterion (Fig. 1d, Table S3, S4), indicating that the suppression of NCI-H1975 cells by ATO alone is sufficient for a therapeutic effect. Neither ATO nor gefitinib treatment satisfied the criterion in A549 cells (Fig. 1d, Table S3, S4).

### ATO mediates autophagy in NSCLC cells

**Fig. 2:**
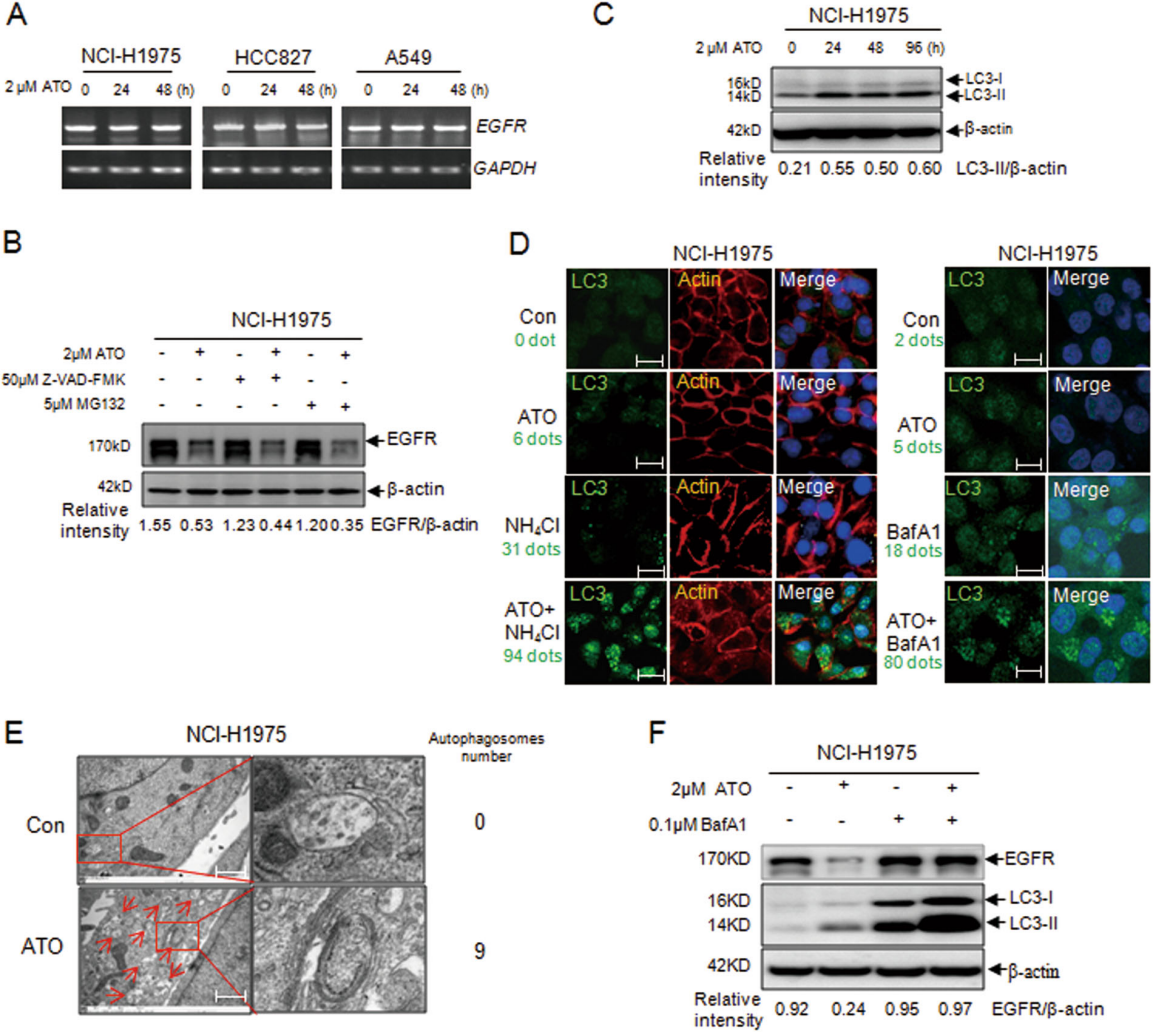
Arsenic-induced autophagy in NSCLC cells. **a** NCI-H1975, HCC827, and A549 cells were treated with ATO for 24 and 48 h, and RT–PCR was performed to detect the transcriptional expression of EGFR. **b** ATO-induced degradation of EGFR in NCI-H1975 cells was assayed in the presence of caspase inhibitor (Z-VAD-FMK) and proteasome inhibitor (MG132). The relative intensity was calculated according to the gray values of EGFR over that of β-actin with the Quantity One software. **c** WB evaluation of the autophagy-related protein LC3-II. The relative intensity was calculated according to the gray values of LC3-II over that of β-actin with the Quantity One software. **d** Expression of LC3 in the NH_4_Cl-pretreated and BafA1-pretreated NCI-H1975 cells were monitored by immunofluorescent assays after incubating with the reagents for 24 h. Images were captured by the LSM710 Pascal software (Scale bars, 15 µm). The immunofluorescent signal has been quantified by counting the number of the green dots (LC3) and showed under the annotation of each groups on the left of the images. **e** Autophagosomes (red arrows) are shown by electron microscopy in NCI-H1975 cells (scale bars, 1 µm). Red framework indicates the magnified sections. The autophagosomes were labeled by red arrows in the Figure. The amount of autophagosomes were quantified by counting the number and displayed on the right of the corresponding image. **f** Validation of the degradation pathway of EGFR. NCI-H1975 cells were treated by ATO with or without BafA1 for 48 h, and the quantity of EGFR was visualized by WB. The relative intensity was calculated according to the gray values of EGFR over that of β-actin with the Quantity One software

ATO showed no effect on EGFR mRNA transcription in all the three NSCLC cell lines (Fig. 2a), indicating a regulation at the protein level. Caspase inhibitor Z-VAD-FMK and proteasome inhibitor MG132 were unable to block the ATO-mediated degradation of EGFR (Fig. 2b), thereby excluding the involvement of these two pathways.

Given that the autophagic pathway is implicated in the action of arsenic^21^, we tested the expression of LC3-II, a characteristic of autophagy activation, in ATO-treated cells. Figure 2c shows that ATO mediated an upregulation of LC3-II in NCI-H1975 cells. The LC3 immunofluorescent staining in both cytoplasm and nucleus slightly increased after ATO treatment in NCI-H1975 cells (Fig. 2d). This increase became more significant when NH_4_Cl or bafilomycin A1 (BafA1) was added to block the protein degradation by proteases in lysosomes and in autophagosomes (Fig. 2d). Moreover, electron microscopy data showed a number of autophagosomes with double layer membrane-wrapped cytoplasmic components in ATO-treated NCI-H1975 cells (Fig. 2e). These observations reinforce the conclusion that ATO induces autophagy in NCI-H1975 cells.

### ATO promotes the autophagic degradation of EGFR regulated by the autophagy receptor P62

The autophagy inhibitor BafA1 significantly inhibited the ATO-mediated degradation of EGFR in NCI-H1975 cells (Fig. 2f). The data further showed that the half-life of EGFR was shortened by ATO (Fig. S2A) in the presence of the cycloheximide (CHX) but prolonged by BafA1 (Fig. S2B).

**Fig. 3:**
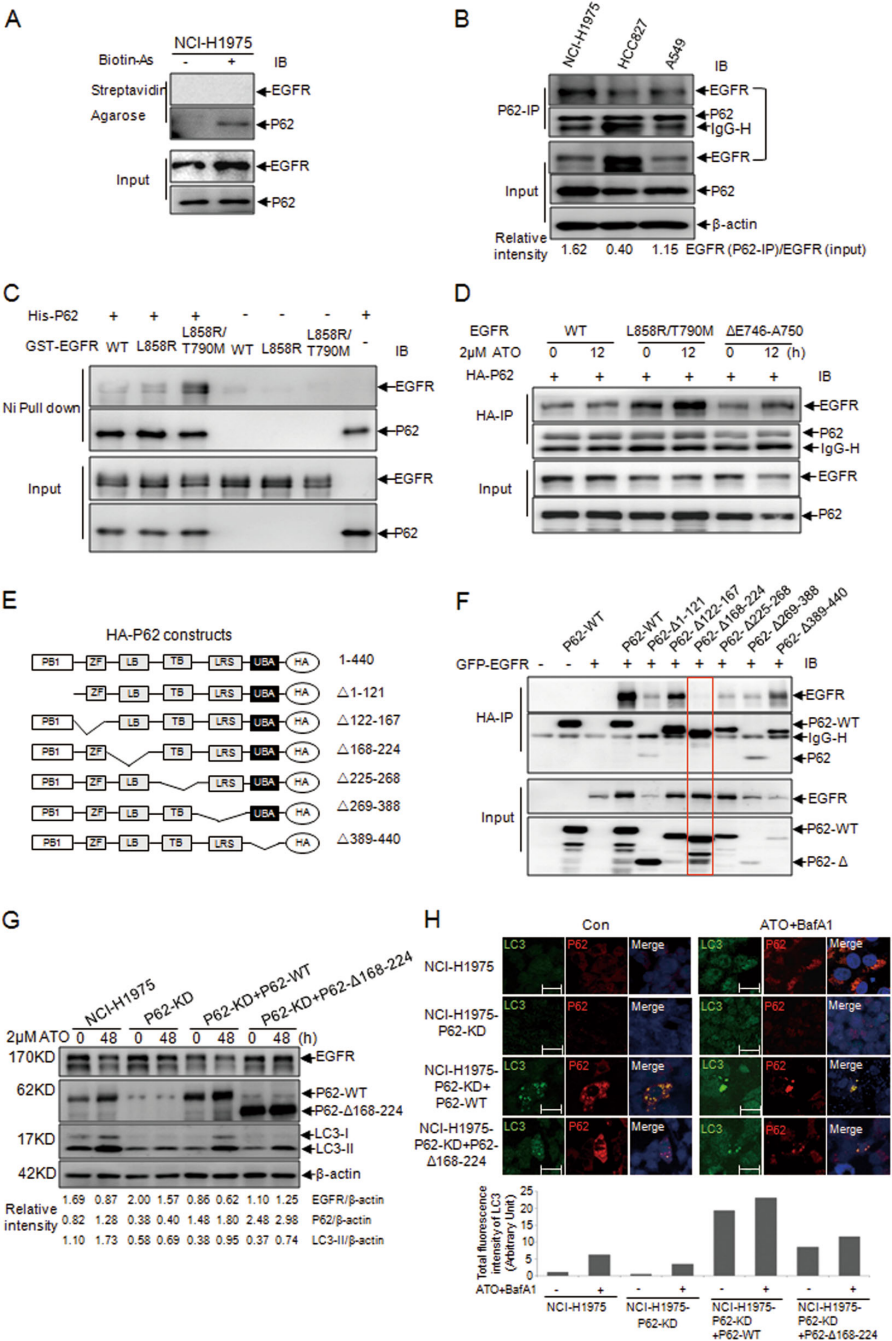
Molecular evidence for the interaction between EGFR and P62. **a** Detection of the interaction between EGFR, P62, and Biotin-As with the streptavidin agarose affinity assay. **b** Demonstration of the intracellular interactions between EGFR and P62 in NCI-H1975, HCC827, and A549 cells by co-IP assays. **c** Pull-down assays for the interaction of the purified His-P62 with the GST-EGFR (WT, L858R, and L858R/T790M). **d** Interaction of ectopically expressed EGFR mutants with wild-type P62 in 293T cells treated with or without 2 µM ATO for 12 h. **e** Schematic representation of the P62 mutational constructs. (PB1 Phox and Bem1 domain, ZF Zinc finger domain, LB LIM protein-binding domain, TB TRAF6-binding domain, LRS LC3-recognition sequence, UBA ubiquitin-associated domain). **f** Mapping the binding domain of P62 and EGFR by co-transfecting WT and mutant P62 and EGFR WT into 293T cells. **g** Detection of the ATO-induced EGFR degradation and the autophagic level (LC3 level) by using WB in P62-KD cells transfected by the P62-WT or P62-Δ168–224. The relative intensity was calculated according to the gray values of EGFR, LC3-II and P62 over that of β-actin with the Quantity One software. The NCI-H1975 cells and the P62-KD cells were as the positive and negative controls, respectively. **h** Immunofluorescence results from the P62-KD cells transfected by P62-WT or P62-Δ168–224. The transfected cells were pretreated with BafA1 (0.1 μM) for 4 h followed by a treatment with 2 μM ATO for 48 h. The expression and distribution of LC3 (green) and P62 (Red) were monitored. The total fluorescence intensity of LC3 was analyzed by using the Bioflux 2000 software and presented under the images. Images were captured by the LSM710 Pascal software (Scale bars, 15 µm). The NCI-H1975 cells and the P62-KD cells were as the positive and negative controls, respectively

To test whether arsenic directly binds to EGFR to initiate autophagy, the streptavidin agarose affinity assay with the biotinylated arsenic (*p*-aminophenylarsine oxide, PAPAO) was performed and showed that it was not the case (Fig. 3a). We then sought to identify the intermediate molecules involved in EGFR degradation.

P62 is the first selective autophagy receptor that mediates the protein degradation through autophagic pathway^22^.The NCI-H1975 cell lines with persistently silenced P62 (P62-KD) were thus established (Fig. S2C) to test the effect of P62 on EGFR degradation. The ATO-induced EGFR degradation was significantly inhibited in NCI-H1975 cells with different P62 siRNA targets (Fig. S2D). On the other hand, the overexpression of P62 in the cells rescued the EGFR degradation (Fig. S2E). These results suggested that P62 participates in ATO-induced EGFR degradation.

### ATO promotes the interaction of P62 with EGFR more substantially with the L858R/T790M mutant

To test whether P62 and EGFR interact with each other, co-immunoprecipitation was performed. Results showed that EGFR indeed interacted with P62 in the three NSCLC cell lines (Fig. 3b, S3A). Interestingly, this interaction was enhanced in the cells bearing the L858R/T790M-mutated EGFR (Figs. 3b, 3d). Consistent results were obtained from the pull-down experiments using purified GST-EGFR (WT, L858R, and L858R/T790M) and His-P62 (Fig. 3c). Importantly, the interaction between P62 and L858R/T790M-mutated EGFR was further enhanced by ATO in the transfected 293T cells (Fig. 3d) and NSCLC cell lines (Fig. S3A). In addition, the L858R/T790M-mutated EGFR underwent an enhanced degradation after ATO treatment (Fig.1c). Data (Fig. 3c, d) further indicated that the amino acid residues, L858 and T790, of EGFR might likely be important in interacting with P62.

Domains within P62 responsible for the EGFR/P62 interaction were mapped. Results showed that P62 with the Δ168–224 deletion (P62-Δ168–224) lost the ability to bind EGFR (Fig. 3e, f, and S3B), suggesting its crucial role in mediating the interaction. Given that P62 binds to poly-ubiquitinated proteins and mediates the autophagic degradation of these proteins^22–24^, we would ask whether the poly-ubiquitination of EGFR is also required for its association with P62 in autophagic degradation. Mutant P62 with a deletion of the UBA domain (389–440) responsible for binding to poly-ubiquitinated proteins was thus tested and results showed an unaffected P62/EGFR interaction (Fig. 3f), strongly suggesting that the EGFR/P62 interaction is unlikely dependent on the ubiquitination on EGFR.

We assumed that P62-Δ168–224 could affect the autophagy level and the degradation of EGFR mediated by ATO. Indeed, results showed that ATO-induced EGFR degradation was significantly inhibited in P62-KD cells which were rescued by P62-WT but not P62-Δ168–224 (Fig. 3g). In the case of LC3, the decreased LC3-II level after ATO treatment in P62-KD cells was rescued by the transfection of P62-WT and weakly by that of P62-Δ168–224 (Fig. 3g). Furthermore, the expression of P62-WT in P62-KD cells resulted in a significantly increased P62 and LC3 signals, and a co-localization of P62 and LC3 was readily identified. When these cells were treated with ATO and BafA1, the intensity of the signals was even further enhanced (Fig. 3h). As for the P62-KD cells transfected with P62-Δ168–224, a slightly weaker intensity of P62 and LC3 signals was displayed in comparison with that in the P62-WT transfected cells (Fig. 3h). These data were basically coincident with those from western blot (Fig. 3g).

### ATO directly binds to P62 through the cysteines in different domains

**Fig. 4:**
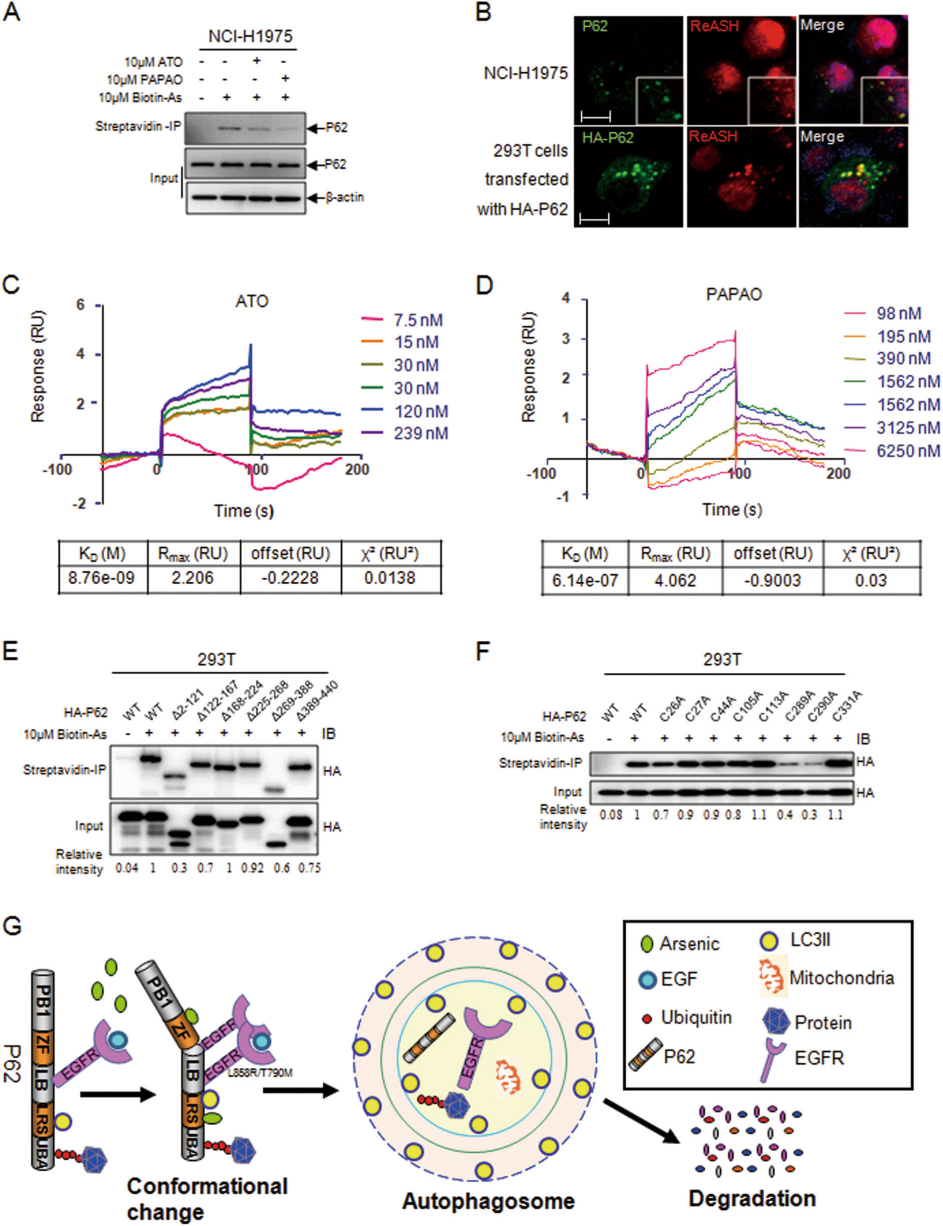
Evidence for arsenic binding to P62. **a** Streptavidin agarose affinity assay for arsenic binding to P62 in NCI-H1975 cells was performed as previously described^12^ with unlabeled arsenic as the competitive control. **b** Co-localization of P62 with the ReAsH in NCI-H1975 and 293T cells transfected with HA-P62 (scale bar, 10 µm). **c** ATO directly binding to P62 was measured by the surface plasmon resonance (SPR) method. Related binding constants are displayed at the down panel. **d** Demonstration of PAPAO binding to immobilized P62 by SPR and the binding constants are shown. **e** Mapping the arsenic binding domains within P62 by streptavidin agarose affinity assay through transfecting WT and mutant P62 into 293T cells. **f** Identification of the cysteines of P62 involved in binding arsenic by substituting alanines for cysteines in P62. **g** Working model for the mechanism by which arsenic binds to P62 to mediate autophagy

We examined the interaction of biotinylated arsenic with P62 and found indeed that arsenic directly bound to P62 in NCI-H1975 cells or in 293T cells transfected with HA-P62 (Fig. 4a, S4A). This binding was competitively inhibited by the unlabeled arsenic (Fig. 4a, S4A). This molecular association was further confirmed by immunofluorescent observations where ReAsH was partially co-localized with P62 (Fig. 4b). SPR assays showed that His-P62 protein bound ATO or PAPAO at *K*_D_ values of 8.76 nM for ATO and 614 nM for PAPAO (Fig. 4c, d). All the data pointed to a mechanism in which arsenic binds to P62 in a direct manner.

The amino acids that are responsible for arsenic binding to P62 were further identified. As showed in Fig. 4e and S4B, the 2–121, ZF (zinc finger) (122–167), and 269–388 domains of P62 were involved in arsenic binding. Alanine substitution experiments showed that the two adjacent cysteines C289 and C290 were required in arsenic binding (Fig. 4f, S4B). Additionally, C128, C131, and C154, except C151 in the ZF1 domain of P62, were also involved in the interaction with arsenic (Fig. S4C, Table S5). These indicate a multiple pattern for the association of arsenic with the cysteines of P62. The mechanism for the arsenic-induced autophagic degradation of EGFR in NSCLC was summarized in a working model (Fig. 4g).

### ATO inhibits tumor growth in NSCLC xenograft mice

**Fig. 5:**
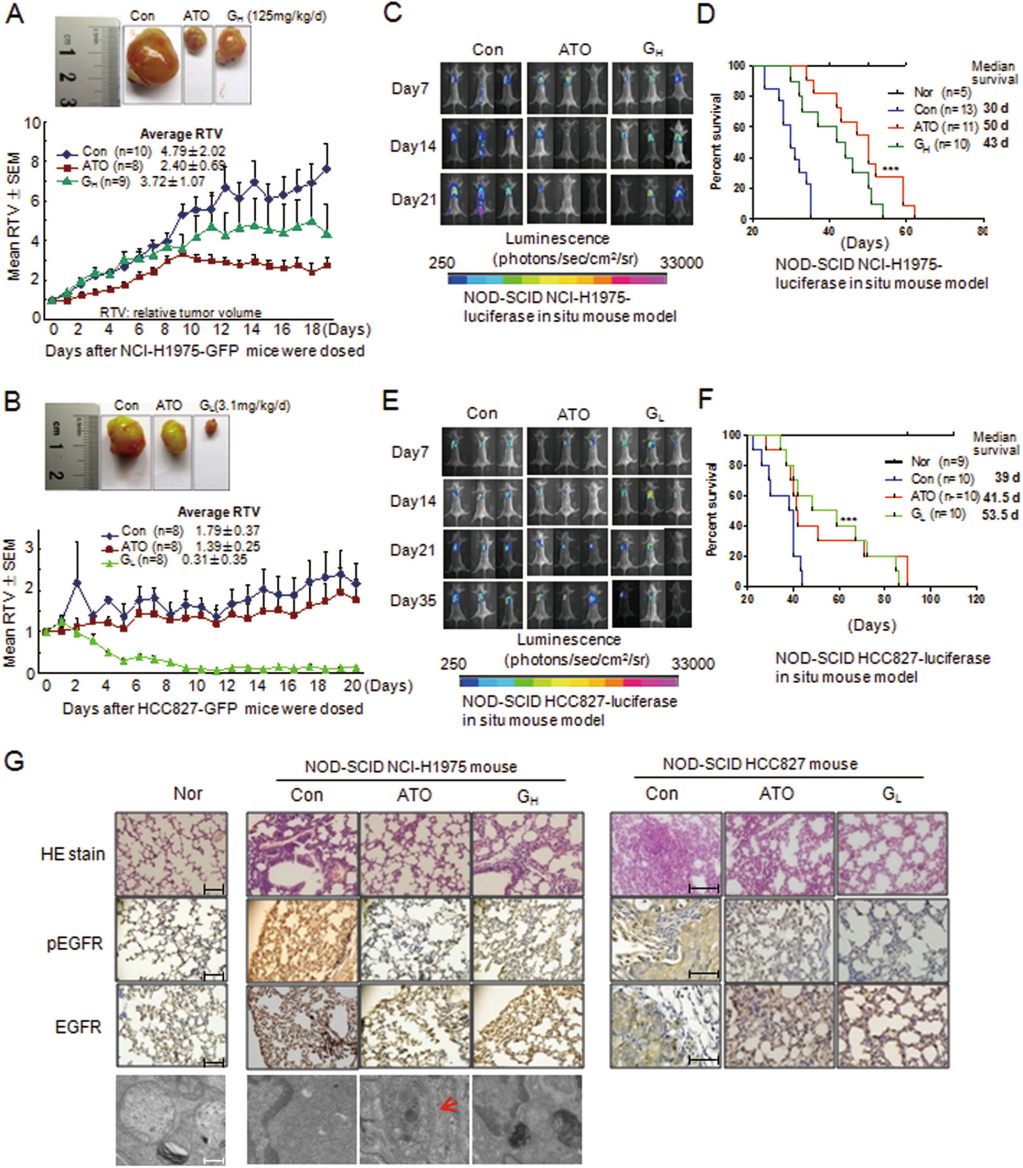
Arsenic and gefitinib inhibit tumor growth in NCI-H1975 and HCC827 xenograft mice in vivo. **a** NCI-H1975-GFP-bearing nude mice were randomized into three groups, i.e., control (Con, treated with sterile water; intragastric administration), ATO (ATO, 5 mg/kg/d; intraperitoneal injection), and gefitinib (G_H_, 125 mg/kg/d; intragastric administration). Representative photographs showing the tumor size were taken on day 18 of treatment (upper panel). Tumor growth curve is shown at the down panel. All values are presented as mean with SEM. RTV is the relative tumor volume. **b** HCC827-GFP-bearing nude mice were randomized into three groups, which were similar to those in NCI-H1975 mice except for the gefitinib group, in which gefitinib was applied at low dose (G_L_, 3.125 mg/kg/d; intragastric administration). Representative photographs showing the tumor size were taken on day 20 of treatment (upper panel). Tumor growth curve is shown at the down panel. All values are presented as mean with SEM. **c** IVIS representative photographs of the NCI-H1975-luciferase in situ mouse models were obtained on days 7, 14, and 21 of treatment. Doses of ATO and gefitinib to treat the in situ models were similar to those in nude mouse models. **d** Survival curves of NCI-H1975 in situ mouse models. ***p < 0.0001 compared with control. Median survival is labeled. The median survival was shown in the right. **e** IVIS representative photographs of the HCC827-luciferase in situ mouse models were obtained on days 7, 14, 21, and 35 of treatment. Doses of ATO and gefitinib to treat in situ models were similar to those in nude mouse models. **f** Survival curves of HCC827 in situ mouse models. ***p < 0.0001 compared with control. The median survival was shown in the right. **g** Results from the HE staining, immunohistochemistry, and electron microscopy of the pulmonary tissues from the NCI-H1975 in situ mouse models. Scale bars represent 100 µm for the pathological and immunohistochemical images and 0.75 µm for electron microscopy images

In the NCI-H1975-bearing nude mice, tumor growth was significantly inhibited by ATO at 5 mg/kg/d (conventional dose for patients with leukemia is 10 mg/kg/d). The average tumor volume after ATO treatment was reduced to ~1/2 (2.40/4.79) compared with that of the control, in contrast to a 1/4 reduction (3.72/4.79) by gefitinib at 125 mg/kg/d (G_H_) that is ~30 times the conventional dose to treat patients with sensitive NSCLC (250 mg/d, roughly 4.2 mg/kg/d) (Fig. 5a, S5A). Electron microscopic images showed the presence of autophagosomes in tumor cells from the ATO-treated mice (Fig. S5C). As a control, in the HCC827-bearing nude mice, as expected, gefitinib at a relatively low dose (G_L_) (3.1 mg/kg/d) induced a significant inhibition on tumor growth, in contrast to a weak, if not trivial, effect of ATO (Fig. 5b, S5B). These data clearly indicated that ATO was effective for the gefitinib-resistant model comparable with gefitinib for the gefitinib-sensitive model, which was consistent with our in vitro observations.

The NOD-SCID-NCI-H1975-leu in situ model showed that the size and intensity of luciferase signals were significantly decreased in the ATO-treated group compared with those in the control, in which the signals were disseminated into the enterocoelia (Fig. 5c). More importantly, survival analysis showed significantly a longer life span in the ATO-treated mice than in the control (Fig. 5d, *p* < 0.0001). Median survival of the ATO-treated mice was extended to ~1.60-fold (50/30). Whereas gefitinib, even at high doses (125 mg/kg/d), was only prolonged by ~1.40-fold (43/30). Again, in the HCC827 model, gefitinib reduced the tumor size, and ATO also had some effect (Fig. 5e). Furthermore, gefitinib significantly prolonged the life span, and so did ATO, even though relatively weakly (Fig. 5f). The median survival of the ATO- or gefitinib-treated mice was 41.5 and 53.5 days, respectively. It is noteworthy that the median survival of the gefitinib-treated mice was prolonged >10 days compared to that of ATO-treated mice. Median survival of the ATO-treated mice was scarcely prolonged by 1.06-fold (41.5/39), which was significantly less than that in the G_L_-treated (3.1 mg/kg/d) mice at 1.37-fold (53.5/39) (Fig. 5f). Notably, prolonged median survival by ATO in the gefitinib-resistant models (1.60-fold) was comparable with that by gefitinib in gefitinib-sensitive models (1.37-fold). Histochemical data showed that the expression levels of EGFR and pEGFR in the lung were downregulated by the treatment of ATO in NCI-H1975 models and by gefitinib in HCC827 models (Fig. 5g). As expected, autophagosomes were detected in the lung of the ATO-treated NCI-H1975 model (Fig. 5g). ATO- and/or gefitinib-treated mice showed no significant adverse effects demonstrated by liver/body weight, spleen/body weight, and serum AST and LDH (Fig. S5D).

## Discussion

High expression and/or gain-of-function mutations of EGFR contribute to NSCLC tumorigenesis. Patients harboring the EGFR-activating mutations benefit from the kinase inhibitor gefitinib but may have to confront the drug resistance caused by the secondary mutations^7,25^. Here we developed a strategy by diminishing the EGFR protein level instead of inhibiting its activity to suppress the overall potential of tumorigenesis of NSCLC. Generally, ATO at 2 µM induced modest quantitative EGFR reductions in HCC827 and A549 cells, whereas a significantly reduction in NCI-H1975 cells (Fig. 1a). To determine whether ATO inhibition is of therapeutic value, we applied the inhibition of overall kinase activity of EGFR achieved by gefitinib in HCC827 as a criterion (Fig. 1d). Only in NCI-H1975 cells, a significant inhibition of the overall kinase activity by ATO met this criterion (Fig. 1d), indicating that ATO may exert an effect on gefitinib-resistant NCI-H1975 cells comparable with that by gefitinib on gefitinib-sensitive HCC827 cells in terms of the tumorigenic potential. We conclude that ATO at an applicable dose effectively suppressed the gefitinib-resistant NCI-H1975 cells as evidenced by a potent inhibition of the overall EGFR kinase activity that met the “gefitinib criterion”.

**Fig. 6:**
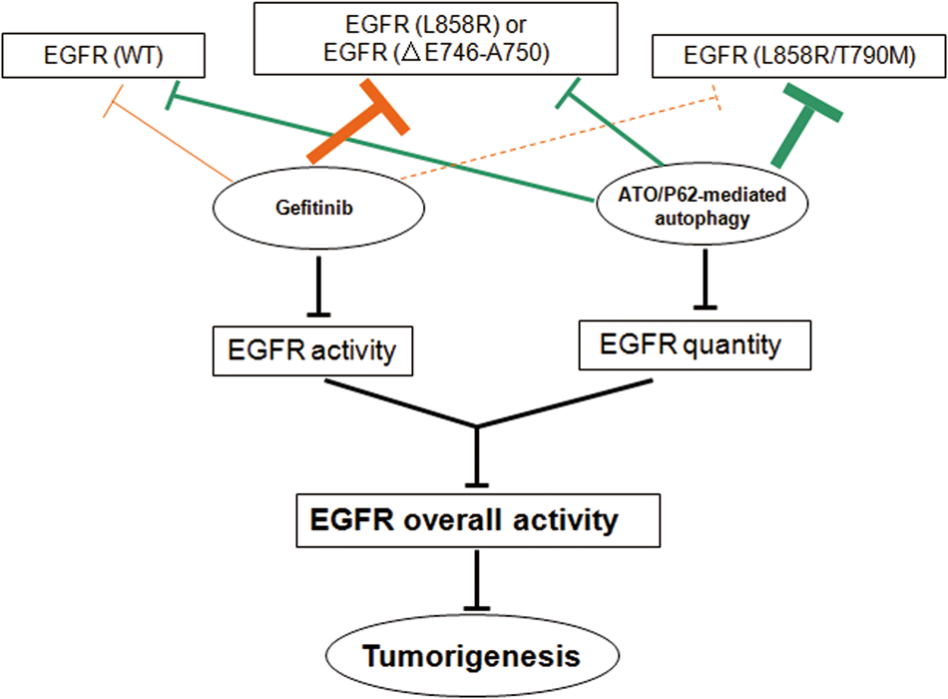
Schematic of the effect of arsenic and gefitinib on the overall EGFR kinase activity in NSCLC. “**⊣**” represents the inhibitory effect (orange colored for gefitinib and green colored for ATO/P62-mediated autophagy). Line thickness represents the inhibition extent. Dotted line indicates the barely detectable inhibition

P62 showed a stronger interaction with the L858R/T790M-mutated EGFR than that with WT EGFR (Fig. 3c, d). The interaction between P62 and EGFR was responsible for the ATO-induced EGFR degradation, evidenced by data from Fig. 3g. Interestingly, arsenic binding to P62 further enhanced this interaction along with a bulk degradation of EGFR (Fig. 1c). The schematic diagram of this molecular regulation is delineated in Fig. 6, suggesting that the therapeutic effect of ATO on NSCLC cells, in which P62, upon ATO binding, might undergo conformational changes that enable an enhanced interaction with the EGFR L858R/T790M mutant and in turn lead to an autophagy-based EGFR degradation (Fig. 4g). However, precise structural data are required for the improved elucidation of the exact mechanisms.

The development of EGFR tyrosine kinase inhibitors progresses rapidly^8,26^. These inhibitors exerted a therapeutic effect by targeting the EGFR kinase region^8,27,28^. However, new resistant mutations tend to emerge following the application of each inhibitor, most likely because of the dominant selection of the clones^28,29^. Even though AZD9291 and EGF816 can overcome gefitinib resistance^9,30,31^, the new mutation C797S accordingly emerges. Confronting this situation, EAI045 was designed to overcome this resistance^8^. Newer drug-resistant mutations are reasonably anticipated and next generation of inhibitors should be designed. Therefore, strategies other than inhibitors, including degradation induction, would help to circumvent this critical issue. As a matter of fact, the action of ATO in the EGFR autophagic degradation was mediated by P62 that interacted with EGFR preferentially the L858R/T790M mutant (Figs. 1c, 3c, d), even though the precise mechanisms are still unclear. Besides a potent effect on the L858R/T790M mutant, there is an inhibition of ATO on wild type or other EGFR mutants even inferior to the “gefitinib criterion”. This feature may probably allow ATO to exert its major effect on gefitinib-resistant EGFR in NCI-H1975 cells simultaneously with a relatively weaker but general inhibition on other types of EGFR (ΔE746-A750 and WT), thereby reducing or preventing the dominant selection of new mutants.

In addition to NSCLC, high levels of EGFR expression also identified in tumors of breast^32^, pancreas^33^, colon^32^, ovary^34^, and brain origins^34^. The mechanism of the effect of ATO on NSCLC cells through a P62/mutant EGFR/autophagy axis allows us to foresee a new paradigm of the roles of arsenic in treating malignancies by targeting the regulatory pathways to promote the turnover of molecules of key pathological importance.

Descriptions regarding the roles of autophagy in tumor biology are controversial. Tumor cells trigger autophagy to achieve self-protection and prevent death^35,36^. On the other hand, they would be killed when autophagy reaches rather advanced levels, particularly when induced by some drugs^35^. The oncogenic EGFR was recently shown to aberrantly bind to Beclin-1, which might contribute to tumor progression and chemoresistance^37^. In this study, ATO upregulated the autophagic marker LC3 (Fig. 2c, d) and increased the number of autophagosomes (Fig. 2e), which finally led to the inhibition of proliferation and cell death (Fig. 1a, S1A). ATO-induced EGFR degradation through the autophagic pathway (Fig. 2f), and the decrease of EGFR could in turn drastically induce autophagy^38,39^. Such regulation with positive feedback will finally lead to extensive cellular autophagy and death.

Previous studies demonstrated that ATO bound to cysteines in the RBCC domain of PML and induced PML-RARα degradation^11–13^. The dicysteine motif (C212 and C213) and cysteines in the RF domain were thought to be involved in arsenic binding^12,40^. In this study, P62 directly bound arsenic (Fig. 4, S4); and the two vicinal cysteines 289 and 290, as well as the ZF domain (122–167), might be implicated in this binding (Fig. 4f, S4B)^41^^,^^42^.

P62 plays an essential role in targeting poly-ubiquitinylated proteins to autophagosome for degradation^43^. In this study, the UBA domain-deleted P62 still interacted with EGFR (Fig. 3f) indicating that the EGFR degradation mediated by P62 mostly occurred in its un-ubiquitinated form (Fig. 3f). This result proposes a unique mechanism whereby ATO directly binds to P62 (Fig. 4), enhancing its interaction with EGFR (Fig. 3d, S3A) and the degradation of the kinase (Fig. 1c).

ATO inhibited tumor growth and prolonged the life span, especially in the mouse models of NCI-H1975 cells (Fig. 5). The in situ model is thought to represent the systematic effect of the reagents more adequately. The effect of ATO on prolonging the life span in the gefitinib-resistant in situ models was comparable with that of gefitinib in the gefitinib-sensitive models of HCC827 cells, which reinforced our conclusion regarding the in vitro effects of ATO on NCI-H1975 and gefitinib on HCC827 cells. In the nude mouse models, the suppression of NCI-H1975 tumor volume by ATO was of sufficient magnitude to account for the similar inhibitory effect of low-dose gefitinib on HCC827 models. These in vivo observations led to a conclusion that ATO at a subtherapeutic dosage exerts a substantial therapeutic effect on the gefitinib-resistant NCI-H1975 cells similar to that of gefitinib on the gefitinib-sensitive HCC827-derived tumor, which was nicely consistent with the in vitro data in the context of the overall EGFR kinase activity.

In short, our observations allow the postulation of an expanded application of ATO to treat NSCLC. We proposed that the reduced overall EGFR kinase activity by either decreasing the quantity by ATO or inhibiting the kinase activity by TKIs might be crucial therapeutic strategies. Moreover, the identification of P62 as a novel ATO target protein and the analysis of the binding specificity provide insights into the precise mechanisms for ATO binding to its target proteins. Further investigation on the structural basis with a wider spectrum of target proteins may increase the significance of arsenic in important biological processes and in the treatment of different malignancies.

## Materials and methods

### Cell lines, antibodies, and reagents

Gefitinib-resistant NCI-H1975 with EGFR L858R/T790M, gefitinib-sensitive HCC827 with EGFR ΔE746-A750, and gefitinib-insensitive A549 with EGFR WT were obtained from ATCC. NCI-H1975-luciferase and HCC827-luciferase cell lines were established by lenti-viral infection with the Ubi-MSC-Luc-firefly-RFP virus purchased from Genechem (Shanghai, China). The NCI-H1975-GFP and HCC827-GFP cell lines were established by lenti-viral infection with pLVX-shRNA2 empty vector (Clonetech, Palo Alto, CA, USA). The NCI-H1975 stable cell lines with persistent silence of P62 were established by lenti-viral infection with pLVX-shRNA2 vector carrying the different siRNA target sequences and were sorted by a MoFlo cell sorter based on ZsGreen. CHO cells were purchased from ATCC and transfected with the WT or mutant EGFR (L858R/T790M and ΔE746-A750), as well as the pEGFP-C1 blank vector. The GFP positive cells were purified by using cell sorter (BD FACSAria III).

The antibodies are shown as follows: EGFR (ab52894), GFP (ab290), and HA (ab9110) were purchased from Abcam (Cambridge, MA, USA). LC3 was obtained from Santa Cruz Biotechnology (Santa Cruz, CA, USA). β-actin was obtained from Sigma-Aldrich (St. Louis, MO, USA), and P62 was from Medical and Biological Laboratories (Nagoya, Japan). GAPDH antibody was the product from Abways Technology (Shanghai, China).

Protein A sepharose beads were purchased from GE Healthcare (Piscataway, NJ, USA). Streptavidin agarose was obtained from G-Biosciences. ReAsH-EDt2 (ReAsH) was obtained from Sigma-Aldrich. Ni sepharoseTM 6 Fast Flow was from GE Healthcare (Chicago, USA). ATO was kindly provided by Beijing Shuang Lu (SL) Pharmaceutical Co., Ltd (Beijing, China). Gefitinib was purchased from Sigma-Aldrich (St. Louis, MO, USA). BafA1 was from Santa Cruz. NH4Cl was from Sigma-Aldrich. Protease inhibitors were from Roche (Bagel, Switzerland).

ATO was prepared as a 2 mM stock solution in phosphate-buffered saline (PBS) at −20 °C. Gefitinib was dissolved in DMSO to produce a stock solution of 10 mM at −20 °C. Then, BafA1 was dissolved in ethanol to make a 200 µM stock solution.

### Plasmid construction and transfection

Plasmids of HA-P62/SQSTM1 and pBABE-puro-EGFR were purchased from Addgene. Deletions and point mutants were constructed by QuickChange® Site-Directed Mutagenesis Kit (Agilent, USA) according to the procedure. Primers were designed by QuickChange® Primer Design Program (Agilent online) and are indicated in Table S6. Specific human P62 siRNA targets in Table S6 are according to previous studies^23,42^. Plasmids were constructed by using the restriction sites BamHI and EcoRI. Detailed information about plasmids and primers can be found in Table S6.

The transfection using lipofect-2000 (Invitrogen, USA) according to the protocol provided by the company was performed as previously described^16^.

### Proliferation and viability assay

NCI-H1975, HCC827, and A549 cells were seeded into individual wells of 96-well plates at 2.0 × 10^4^ cells/mL to 2.5 × 10^4^ cells/mL (100 µL per well) in triplicate. After treatment with 0–10 µM gefitinib and/or 0–2 µM ATO for 24 and 48 h at 37 °C, the viability of cells were measured using the Cell Counting Kit-8 (CCK-8) assay (Dojindo Laboratories, Kumamoto, Japan).

### Assay of EGFR tyrosine kinase activity

NCI-H1975, HCC827, and A549 cells were treated with ATO and/or gefitinib at the indicated concentrations. At 24 and 48 h, the cells were collected and lysed in IP buffer (50 mM Tris, pH 8.0; 150 mM NaCl; and 0.5% NP-40) with protease inhibitor. EGFR was purified using anti-EGFR antibody, and its tyrosine kinase activity was measured with tyrosine kinase assay kit (Millipore). Protein concentrations were detected by BCA assay. Biotinylated (Gly)4-Tyr peptide (0.1 mg/mL in 100 mM sodium bicarbonate buffer, pH 8.0) was incubated for 2 h in streptavidin-coated 96-well plates. Non-specific binding sites were blocked by 3% BSA. Tyrosine phosphorylation was initiated by adding the purified EGFR (10 µg for each sample) in 100 µL of assay buffer (20 mM Tris, pH 7.4; 10 mM MgCl_2_; 1 mM MnCl_2_; 0.2 mM ATP; 1 mM dithiothretiol; 25 mM β-glycerol phosphate; 1 mM sodium orthovanadate; and 5 mM EGTA), and the samples were incubated for 30 min at 37 °C^43^. Different concentrations of F-PEG6-IPQA were added as positive controls to assess the dose-dependent kinase activity. Phospho-tyrosine residues were detected by incubation with anti-phospho tyrosine HRP-conjugated antibodies for 2 h. Tetramethylbenzedine was added into the systems, and the absorbance at 450 nm was measured using microplate reader Safire (Tecan, Research Triangle Park, NC, USA).

### Western blot analysis

The cells were collected and lysed in 200 µL of RIPA (50 mM Tris, pH 8.0; 150 mM NaCl; 0.1% SDS; 0.5% sodium deoxycholate; and 1% NP-40) with protease inhibitor. Protein concentrations were measured by BCA assay. Total protein (100 µg) was loaded into the well of 8–15% sodium dodecyl sulfate (SDS)–polyacrylamide gel. After electrophoresis, proteins were transferred to polyvinylidene difluoride (PVDF) membranes (GE Healhcare), probed with the indicated antibodies, visualized by chemiluminescent HRP Substrate (Millipore), and imaged using ImageQuant LAS-4000 (FujiFilm, Tokyo, Japan).

### Immunofluorescence assay

NCI-H1975 cells pretreated with the lysosome inhibitor NH4Cl (20 mM) or autophagy inhibitor BafA1(0.1 μM) for 4 h were grown on coverslips in 24-well plates, then treated with ATO (2 μM) for 24 h, and fixed in 4% paraformaldehyde, permeabilized with 0.2% Triton X-100, blocked with 1% BSA, and incubated with anti-LC3 antibody and actin stain. Images were captured with a laser scanning confocal microscope with LSM710 Pascal software (Zeiss, Jena, Germany). For the rescue assay, plasmids encoding WT or Δ168–224 P62 were transfected into the NCI-H1975 cells in which P62 was persistently silenced (P62-KD). The cells were then pretreated with BafA1 (0.1 μM) for 4 h followed by an ATO treatment at 2 μM for 48 h. Immunofluorescence assay was performed as above described.

293T cells were transfected with HA-P62 for 48 h, together with the NCI-H1975 cells, and incubated with 5 µM ReAsH for 3 h. Immunofluorescence analysis was performed as previously described^16^.

### Electron microscopy

NCI-H1975 cells were treated with ATO for 48 h together with the tissues from mice, fixed in 2% glutaraldehyde made up in 0.1 M PBS (pH 7.2), postfixed in 1.0% OsO4, dehydrated in a progressive ethanol and acetone solution, embedded in Epoxy 618, sectioned using the LKB V-type ultramicrotome, stained with uranyl acetate followed by lead citrate, and observed with PHILIPS CM-120 transmission electron microscope (Philips in the Netherlands). Images were then captured.

### Immunoprecipitation

293T cells were co-transfected with GFP-EGFR and HA-P62 with WT or mutants. At 48 h after transfection, the cells were collected and lysed in IP buffer (50 mM Tris, pH 8.0; 150 mM NaCl; and 0.5% NP-40) with protease inhibitor. Immunoprecipitation was performed by incubating the cell lyses with HA or GFP antibodies overnight at 4 °C and incubating with protein A beads for another 4 h. After washing with 1X PBS, the beads were resuspended with SDS–PAGE loading buffer and boiled for 10 min. Immunoblotting was performed using HA and GFP antibodies as indicated. For NCI-H1975, HCC827, and A549 cells, the co-IP was performed using P62 or EGFR antibody, and the procedure was the same as that for the 293T cell.

### Ni-bead pull-down

Purified EGFR with the tyrosine kinase domain (residue: 696 to end) (EGFR WT, EGFR L858R, and EGFR L858R/T790M) conjugated at the N-terminal with GST tag was purchased from Millipore, and the purified His-P62 (residue: 1–356) was obtained from Abcam. For the pull-down assay, the complex with GST-EGFR and His-P62 were incubated with Ni-beads overnight at 4 °C. After washing with a buffer that included imidazole, the beads were resuspended in SDS–PAGE loading buffer, and the P62 and EGFR were detected by using the indicated antibodies.

### Streptavidin agarose affinity assay

Biotin-As synthesis was performed by the conjugation of PAPAO to activated PFP-Biotin based on a previous study^12,16^. For streptavidin agarose affinity assay, 293T cells transfected with HA-P62 and its mutants or NCI-H1975 cells were treated with 10 µM Biotin-As for 2 h with or without pretreatment using the competed unlabeled arsenic (ATO, PAPAO, and As_4_S_4_) at 10 µM for 2 h. Cells were collected and lysed in 8 M urea buffer. Cell lysates were incubated with streptavidin agarose overnight at 4 °C. After washing with urea buffer, streptavidin agarose were resuspended in SDS–PAGE loading buffer for western blot analysis.

### Surface plasmon resonance (SPR) assay

SPR measurement was performed using a Biacore T200 with four-flow channels and a sensor chip CM5 with dextran matrix. The His-antibody was immobilized on the CM5 chip by using the His capture kit according to the protocol. P62 protein (100 µg/mL) with His-tag was captured by His-antibody for 300 s at the flow rate of 5 µL/min to achieve a capture level of ~1400 RU. For the binding assay, the reaction temperature was controlled at 25 ± 0.1 °C, and the flow rate was set at 30 µL/min. Response obtained from the detection channel (Fc4) was normalized by subtracting the signal simultaneously acquired from the control channel (Fc3), which could eliminate non-specific binding and buffer-induced bulk refractive index changes. PBS buffer was used as running buffer. Different concentrations of ATO and PAPAO were prepared and measured in SPR assay. Sample solutions were injected onto the chip surface for 120 s. After each binding reaction, further dissociation time of 120 s was applied after each injection. Given that the dissociation between the P62 protein and analytes was rapid and complete, no additional regeneration step occurred. Moreover, the captured P62 protein can be regenerated from the chip surface with Glycine-HCl buffer (pH 1.5) for 60 s. Binding affinity between the P62 protein and analytes was fitted in the affinity model with Biacore T200 Evaluation Software 1.0.

### Xenograft NSCLC mouse model and ATO/gefitinib treatment regimen

Experimental procedures performed on mice were approved by the Studies Ethics Committee of Ruijin Hospital, Shanghai Jiao Tong University (permit number: SYXK2011-0113). Surgical procedures involving mice were in accordance with the Institutional Animal Care and Use Committee guidelines.

Nude mouse models were established by subcutaneous inoculation of NCI-H1975-GFP cells or HCC827-GFP cells (7 × 10^6^) in 4–6-week-old mice. Length and width axes of the tumors were measured by calipers daily, and the tumor volumes (mm^3^) were calculated according to the following formula: [(width^2^ × length)/2]. When the volume reached ~100 mm^3^, the mice were randomized into three treatment groups. For NCI-H1975-derived mice, the groups were control (Con, treated with sterile water; intragastric administration), ATO (ATO, 5 mg/kg/d; intraperitoneal injection), and gefitinib (G_H_, 125 mg/kg/d; intragastric administration) (AstraZeneca, Macclesfield, UK, which was dissolved in sterile distilled water). For HCC827-derived mice, the groups were control (Con, treated with sterile water; intragastric administration), ATO (ATO, 5 mg/kg/d; intraperitoneal injection), and gefitinib at low dose (G_L_, 3.125 mg/kg/d; intragastric administration). The relative tumor volume (RTV) was calculated according to the formula: RTV = TV_*n*_/TV_0_, where TV_*n*_ is the tumor volume at day *n*, and TV_0_ is the tumor volume at day 0.

In situ tumor xenografts were established by injecting the NCI-H1975-luciferase cells or HCC827-luciferase cells (8 × 10^6^) to the lungs of the NOD-SCID mice (6–8 weeks) through the intercostal space during surgical operation under general anesthesia. Doses of ATO and gefitinib to treat in situ mice were the same as those in nude mice. Xenograft tumors and pulmonary tissue were excised from killed mice and fixed in formalin or 2% glutaraldehyde for pathology, immunohistochemistry, and electron microscopy assays.

### Small-animal in vivo imaging system (IVIS) analysis

Live-animal tumor imaging was performed after treatment with ATO and/or gefitinib using IVIS (Lumat 9507, Berthod, Germany).

### Immunohistochemistry

Tumor or pulmonary tissues from NSCLC xenograft mice were fixed in formalin and embedded in paraffin. Samples were blocked and incubated with specific antibodies after removal of paraffin and were detected by peroxidase/DAB detection kit (Gene Tech, Shanghai, China). All sections were counterstained with hematoxylin.

### Statistical analysis

The data were reported as mean value with the standard error of mean quantified from at least three independent experiments. Differences were compared using two-tailed Student’s *t*-test. *p*-values < 0.05 were considered statistically significant. All statistical calculations were carried out using GraphPad Prism 5 (San Diego, CA, USA) or Excel (Microsoft, Redmond, USA).

## Electronic supplementary material

supplemental materials

Supplemental Figure 1

Supplemental Figure 2

Supplemental Figure 3

Supplemental Figure 4

Supplemental Figure 5
