# Supplementary material for: Arsenic circumvents the gefitinib resistance by binding to P62 and mediating autophagic degradation of EGFR in non-small cell lung cancer
Source: Cell Death Dis. 2018 Sep 20;9(10):963. doi: 10.1038/s41419-018-0998-7 (PMC6147786; doi:10.1038/s41419-018-0998-7)
Supplement: Supplementary file 1 — supplemental materials [file 41419_2018_998_MOESM1_ESM.doc]

**Supplementary information**

**Arsenic circumvents the gefitinib resistance by binding to P62 and mediating autophagic degradation of EGFR in non-small cell lung cancer**

**Running Title: Arsenic binding to P62 induces autophagy in NSCLC**

Jianhua Mao^1,2,#^, Lie Ma^1,3,#^, Yan Shen^4^, Kongkai Zhu^5^, Ru Zhang^1^, Wenda Xi^6^, Zheng Ruan^1,2^, Cheng Luo^5^, Zhu Chen^1^, Xiaodong Xi^1,2,*^, Saijuan Chen^1,7,*^

^1^State Key Laboratory of Medical Genomics, Shanghai Institute of Hematology, Ruijin Hospital Affiliated to Shanghai Jiao Tong University School of Medicine, 197 Ruijin Road II, Shanghai 200025, China

^2^Collaborative Innovation Center of Hematology, Ruijin Hospital Affiliated to Shanghai Jiao Tong University School of Medicine, 197 Ruijin Road II, Shanghai 200025, China

^3^Shanghai Center for Systems Biomedicine, Shanghai Jiao Tong University, Shanghai 200240, China

^4^Research Center for Experimental Medicine, Ruijin Hospital Affiliated to Shanghai Jiao Tong University School of Medicine, 197 Ruijin Road II, Shanghai 200025, China

^5^Drug Discovery and Design Center, State Key Laboratory of Drug Research, Shanghai Institute of Materia Medica, Chinese Academy of Sciences, Shanghai 201203, China

^6^Shanghai Institute of Hypertension, Ruijin Hospital Affiliated to Shanghai Jiao Tong University School of Medicine, Shanghai 200025, China

^7^Lead Contact

^#^ Authors contributed equally to this work.

*Corresponding Authors: Xiaodong Xi, Shanghai Institute of Hematology, Ruijin Hospital Affiliated to Shanghai Jiao Tong University School of Medicine, 197 Ruijin Road II, Shanghai 200025, China. Phone:+86-21-34187218; Fax:+86-21-64743206; [xixiaodong@shsmu.edu.cn](mailto:xixiaodong@shsmu.edu.cn) or Saijuan Chen, Shanghai Institute of Hematology, Ruijin Hospital Affiliated to Shanghai Jiao Tong University School of Medicine, 197 Ruijin Road II, Shanghai 200025, China. Phone: +86-21-64377859; Fax:+86-21-64743206; [sjchen@stn.sh.cn](mailto:sjchen@stn.sh.cn).

**Disclosure of Potential Conflicts of Interest**

The authors declare no potential conflicts of interest.

**AUTHOR CONTRIBUTIONS**

J.M. helped design the project, designed the experiments, performed the experiment work, collected and analyzed the data, assembled the figures, and wrote the manuscript. L.M. performed the experimental work, collected and analyzed the data, and assembled some figures. Y.S., K.Z., R.Z. and Z.R. performed experimental work. C.L. helped analyzed some data. W.X. helped revised the manuscript. Z. C., X. X., and S. C., helped design the experiments, analyzed the data, wrote and revised the manuscript.

**This file includes**

Figure legends

Figure S1 to S5

Table S1 to S6

**Supplementary Figure legends**

**Figure S1. Proliferation inhibition by ATO and gefitinib in NSCLC cell lines and CHO cells transfected with EGFR WT or mutants, and tyrosine kinase activity of the commercial purified EGFR WT.** A) (a) NCI-H1975, HCC827, and A549 cells were treated with ATO, gefitinib (G), or both (ATOG) for 24 and 48 h. Proliferation inhibition was measured using CCK-8. Data are shown as mean value with SEM. (*, *p*<0.05 versus group G; **, *p*<0.01 versus group G; ***, *p*<0.001 versus group G). (b) CHO cells were transfected with EGFR WT or mutants, as well as the pEGFP-C1 blank vector, after 48h of transfection, the cells were sorted by using GFP antibody by BD AiraIII flow cytometry. The GFP positive cells were seeded on the 96-well plates and treated with ATO, G or both (ATOG) for 48h. Proliferation inhibition was measured using CCK-8. Data are shown as mean value with SEM. B) Tyrosine kinase activity (OD450) of different concentrations of commercial purified EGFR WT.

**Figure S2. Detection of half-life of EGFR and molecular evidence for P62 participating in the degradation of EGFR induced by ATO.** A) Half-life of EGFR was detected using cycloheximide (CHX, 100 µg/mL) alone or in combination with ATO (2 µM). The relative intensity was calculated according to the gray values of EGFR over that of β-actin with the Quantity One software. B) Half-life of EGFR was detected using CHX (100 µg/mL) alone or in combination with BafA1 (0.1 µM). The relative intensity was calculated according to the gray values of EGFR over that of β-actin with the Quantity One software. C) RT-PCR assay was performed to detect the silence of P62 at mRNA level in NCI-H1975 cells. D) Expression levels of EGFR and P62 in NCI-H1975 cells infected with lentivirus carrying different P62 siRNA targets and treated with ATO for 24 and 48 h. Ratios of the gray value of EGFR/β-actin are shown in the right panel. E) Degradation of EGFR induced by ATO was rescued when the P62-WT was enforcedly expressed in P62-KD cells. Ratios of the gray value of EGFR/β-actin are shown on the right panel.

**Figure S3. Molecular evidence for the interaction between EGFR and P62.** A) Demonstration of interaction between EGFR and P62 in NCI-H1975, HCC827, and A549 cells with or without ATO treatment for 12 h using EGFR and P62 antibody. B) Interaction of different P62 mutants with wild-type EGFR in 293T cells. Co-IP was performed using GFP antibody.

**Figure S4. Evidence for arsenic binding to P62 through ZF domain.** A) Streptavidin agarose affinity assay for arsenic binding to P62 in 293T cells transfected with HA-P62 was performed as previously described^16^ with unlabeled arsenic as the competitive control. B) Detection of the binding between P62-ΔZF, C289A, C290A, and Biotin-As. C) Interaction between P62 mutants with different cysteine substitutions in ZF and Biotin-As.

**Figure S5. Photos of the tumor from the NCI-H1975 and HCC827 xenograft nude mice. Detection of the autophagosomes in NCI-H1975 xenograft nude mice and side effects of arsenic and gefitinib on the treated mice.** A) Photos of the tumor from the NCI-H1975-GFP xenograft nude mice. B) Photos of the tumor from the HCC827-GFP xenograft nude mice. C) Electron microscopic photographs demonstrate the autophagosomes in tumor tissues of NCI-H1975 nude mice treated with ATO and/or gefitinib (scale bar 2 µm). D) Liver/body and spleen/body weight ratios of NCI-H1975 in situ mouse models treated with ATO and/or gefitinib were calculated when the mice were sacrificed. Serum AST and LDH of NCI-H1975 in situ mouse models treated with ATO and/or gefitinib were tested using the assay kit according to the provided protocol.

**Table S1**: Calibrated EGFR tyrosine kinase activity based on EGFR WT in A549

|  | NCI-H1975 | | HCC827 | | A549 | |
| --- | --- | --- | --- | --- | --- | --- |
|  | 24h | 48h | 24h | 48h | 24h | 48h |
| Con | 3.063  2.430  2.747 | 3.063  2.740  2.430 | 2.633  2.527  2.630 | 2.890  2.621  2.880 | 1.665  1.535  1.597 | 1.656  1.598  1.534 |
| ATO | 2.188  2.046  1.904 | 1.722  1.693  1.662 | 1.759  1.433  1.621 | 1.707  1.380  1.040 | 1.240  1.370  1.314 | 1.220  1.290  1.330 |
| G | 1.762  1.890  1.999 | 1.624  1.643  1.659 | 0.838  0.816  0.832 | 0.632  0.781  0.719 | 0.912  0.691  0.832 | 0.620  0.750  0.694 |

**Table S2**: Gray value of EGFR in NSCLC cells with or without ATO treatment

|  | Gray value | Ratio to WT |
| --- | --- | --- |
| A549-Con | 1.47 | 1 |
| A549-ATO-24h | 0.81 | 0.55 |
| A549-ATO-48h | 0.68 | 0.46 |
| NCI-H1975-Con | 0.92 | 0.62 |
| NCI-H1975-ATO-24h | 0.49 | 0.33 |
| NCI-H1975-ATO-48h | 0.28 | 0.19 |
| HCC827-Con | 1.74 | 1.18 |
| HCC827-ATO-24h | 1.49 | 1.01 |
| HCC827-ATO-48h | 0.75 | 0.51 |

**Table S3:** Overall EGFR kinase activity of the average of EGFR in NSCLC cells

|  | NCI-H1975 | | HCC827 | | A549 | |
| --- | --- | --- | --- | --- | --- | --- |
|  | 24h | 48h | 24h | 48h | 24h | 48h |
| Con | 1.7162 | 1.7162 | 3.0716 | 3.3084 | 1.5962 | 1.5962 |
| ATO | 0.6929 | 0.3255 | 1.6274 | 0.7020 | 0.7195 | 0.5865 |
| G | 1.1771 | 1.0259 | 0.9805 | 0.8409 | 0.8122 | 0.6909 |

**Table S4:** Inhibition rate of EGFR overall kinase activity (%)

|  | NCI-H1975 | | HCC827 | | A549 | |
| --- | --- | --- | --- | --- | --- | --- |
|  | 24h | 48h | 24h | 48h | 24h | 48h |
| ATO | 66.66 | 84.33 | 50.92 | 80.34 | 54.92 | 63.25 |
| G | 43.37 | 50.64 | 70.43 | 76.45 | 49.11 | 56.71 |

**Table S5:** Bond lengths between arsenic or zinc and the conserved cysteine/histidine residues of P62.

|  | P62-ZF1 | | | | P62-ZF2 | | | |
| --- | --- | --- | --- | --- | --- | --- | --- | --- |
| Conserved Residues | Cys128 | Cys131 | Cys151 | Cys154 | Cys142 | Cys145 | His160 | His163 |
| Zn-P62(Å) | 2.38 | 2.39 | 2.45 | 2.35 | 2.30 | 2.31 | 2.19 | 2.22 |
| As-P62(Å) | 2.31 | 2.29 | 3.43 | 2.27 | / | / | / | / |

**Table S6:** Information of the plasmids and primers used in this study

| **Plasmid name** | **Source** | | **vector** | **Forward primer /**  **siRNA target sequence** |
| --- | --- | --- | --- | --- |
| HA-P62/SQSTM1 | Addgene | pcDNA4/TO | | / |
| pBABE-puro-EGFR WT | Addgene | pBABE-puro | | / |
| HA-P62-ΔZF | Reconstruct | pcDNA4/TO | | gccccgcaacatgttccccagcccct |
| HA-P62-C128A | Reconstruct | pcDNA4/TO | | gcaccccaatgtgatcgccgatggctgcaatggg |
| HA-P62-C131A | Reconstruct | pcDNA4/TO | | gtgatctgcgatggcgccaatgggcctgtggt |
| HA-P62-C142A | Reconstruct | pcDNA4/TO | | gaacccgctacaaggccagcgtctgcccag |
| HA-P62-C145A | Reconstruct | pcDNA4/TO | | ctacaagtgcagcgtcgccccagactacgacttg |
| HA-P62-C151A | Reconstruct | pcDNA4/TO | | ccagactacgacttggctagcgtctgcgaggg |
| HA-P62-C154A | Reconstruct | pcDNA4/TO | | cgacttgtgtagcgtcgccgagggaaagggcttg |
| HA-P62-H160A | Reconstruct | pcDNA4/TO | | gggaaagggcttggcccgggggcacacc |
| HA-P62-H163A | Reconstruct | pcDNA4/TO | | cttgcaccggggggccaccaagctcgca |
| HA-P62-Δ1-122 | Reconstruct | pcDNA4/TO | | tccagtgtggtggaattctgtgcaccccaatgt |
| HA-P62-Δ168-224 | Reconstruct | pcDNA4/TO | | ggcacaccaagctcgcagcttctggtccatc |
| HA-P62-Δ225-268 | Reconstruct | pcDNA4/TO | | gccccacggcagaatcaacccccgtc |
| HA-P62-Δ269-388 | Reconstruct | pcDNA4/TO | | aagaagccgcctggaggctgacccgc |
| HA-P62-Δ389-440 | Reconstruct | pcDNA4/TO | | ccacatctcccgccacgcggccg |
| HA-P62-C26A | Reconstruct | pcDNA4/TO | | ccgcttcagcttcgcctgcagccccgag |
| HA-P62-C27A | Reconstruct | pcDNA4/TO | | gcttcagcttctgcgccagccccgagcctg |
| HA-P62-C44A | Reconstruct | pcDNA4/TO | | ggtccgggacccgccgagcggctgct |
| HA-P62-C105A | Reconstruct | pcDNA4/TO | | acattaaagagaaaaaagaggcccggcgggaccaccg |
| HA-P62-C113A | Reconstruct | pcDNA4/TO | | ccaccgcccaccggctgctcaggaggcg |
| HA-P62-C289A | Reconstruct | pcDNA4/TO | | cacagccaagcagcgcctgctctgacccca |
| HA-P62-C290A | Reconstruct | pcDNA4/TO | | gccaagcagctgcgcctctgaccccagc |
| HA-P62-C331A | Reconstruct | pcDNA4/TO | | acagatggagtcggataacgcttcaggaggagatgatgac |
| pLVX- P62-shRNA1 | Reconstruct | pLVX-shRNA | | gcattgaagttgatatcgat |
| pLVX- P62-shRNA2 | Reconstruct | pLVX-shRNA | | cagatggagtcgggaaact |
| pLVX- P62-shRNA3 | Reconstruct | pLVX-shRNA | | ggttgacattgatgtggaaca |
| pEGFP-EGFR | Reconstruct | pEGFP-C1 | | / |
| pEGFP-EGFR-ΔE746-A750 | Reconstruct | pEGFP-C1 | | tcccgtcgctatcaagacatctccgaaagcca |
| pEGFP-EGFR-L858R | Reconstruct | pEGFP-C1 | | tcacagattttgggcgggccaaactgctggg |
| pEGFP-EGFR-T790M | Reconstruct | pEGFP-C1 | | ccaccgtgcaactcatcatgcagctcatgc |
| pEGFP-EGFR-L858R/T790M | Reconstruct | pEGFP-C1 | | / |
