# Supplementary figures and images for: Arsenic circumvents the gefitinib resistance by binding to P62 and mediating autophagic degradation of EGFR in non-small cell lung cancer
Source: Cell Death Dis. 2018 Sep 20;9(10):963. doi: 10.1038/s41419-018-0998-7 (PMC6147786; doi:10.1038/s41419-018-0998-7)

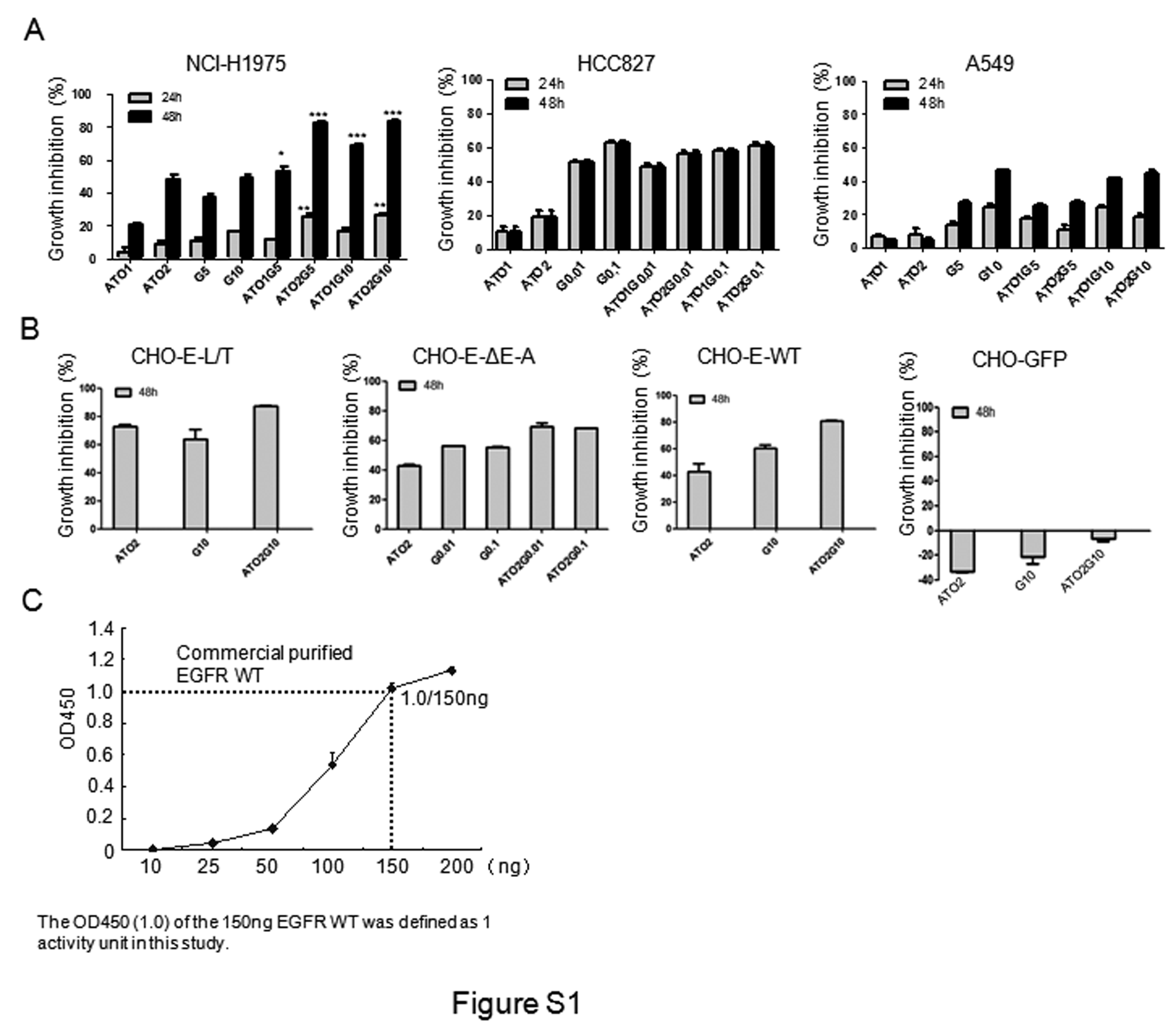

Supplement: Supplementary file 2 — Supplemental Figure 1 [file 41419_2018_998_MOESM2_ESM.tif]

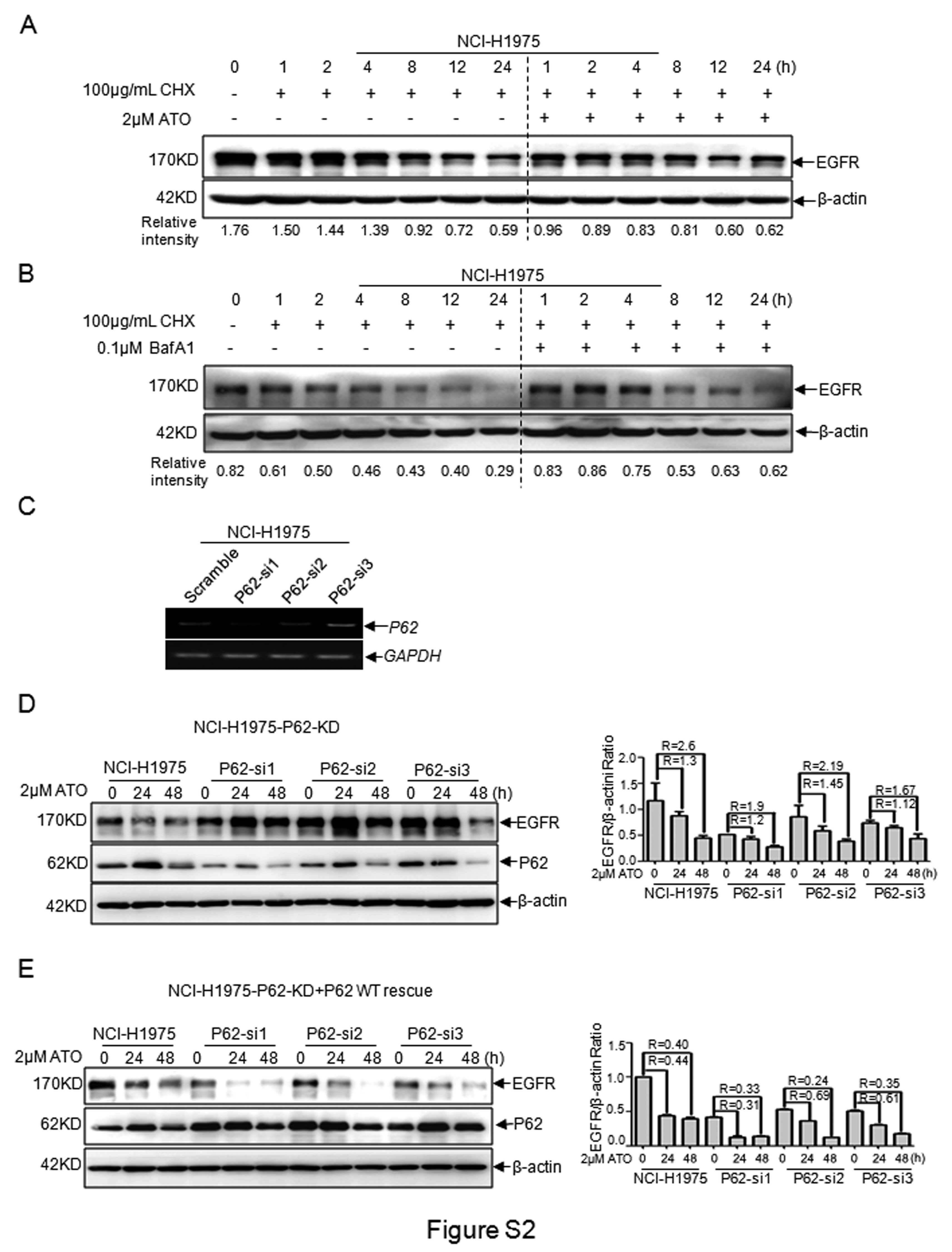

Supplement: Supplementary file 3 — Supplemental Figure 2 [file 41419_2018_998_MOESM3_ESM.tif]

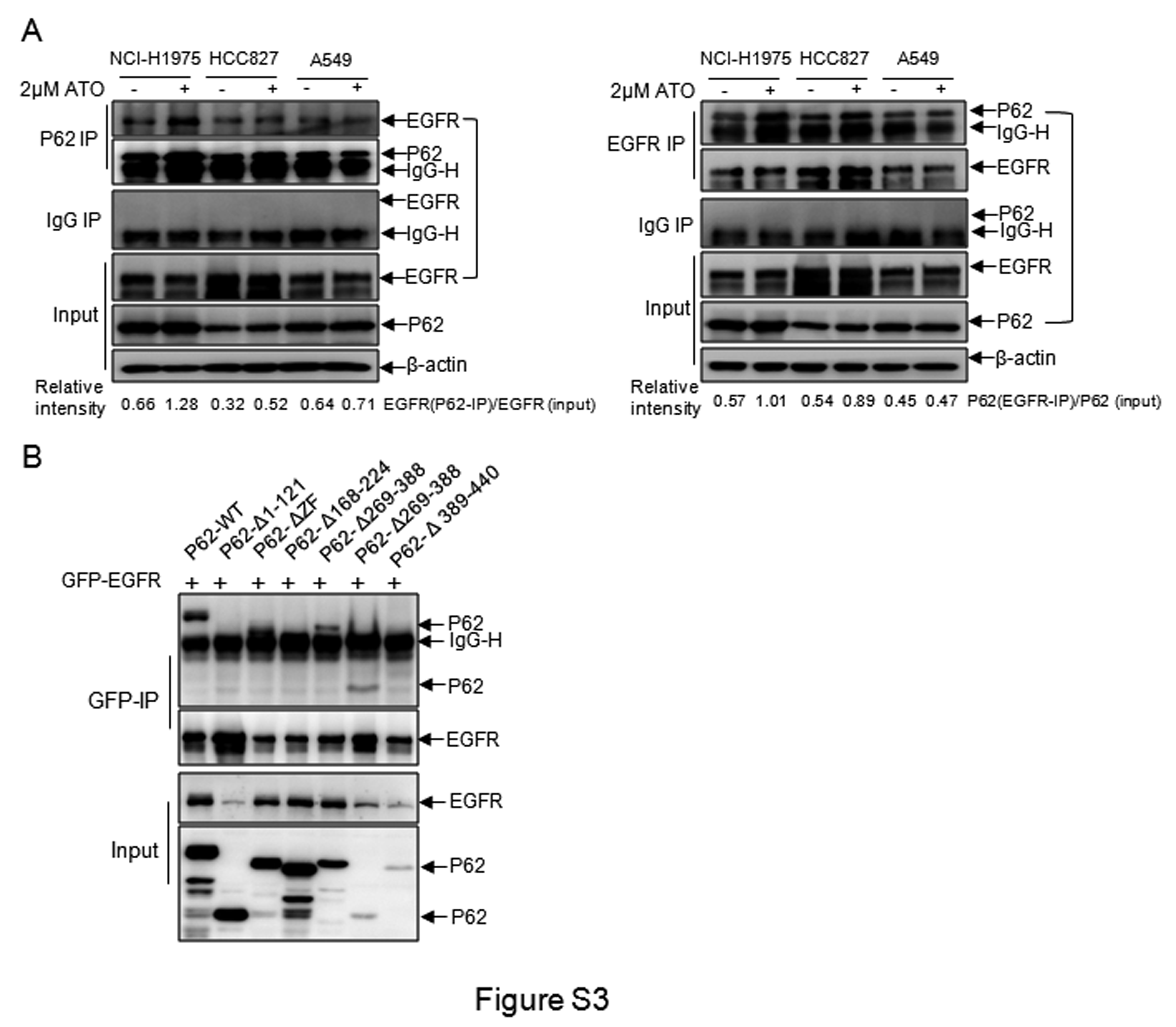

Supplement: Supplementary file 4 — Supplemental Figure 3 [file 41419_2018_998_MOESM4_ESM.tif]

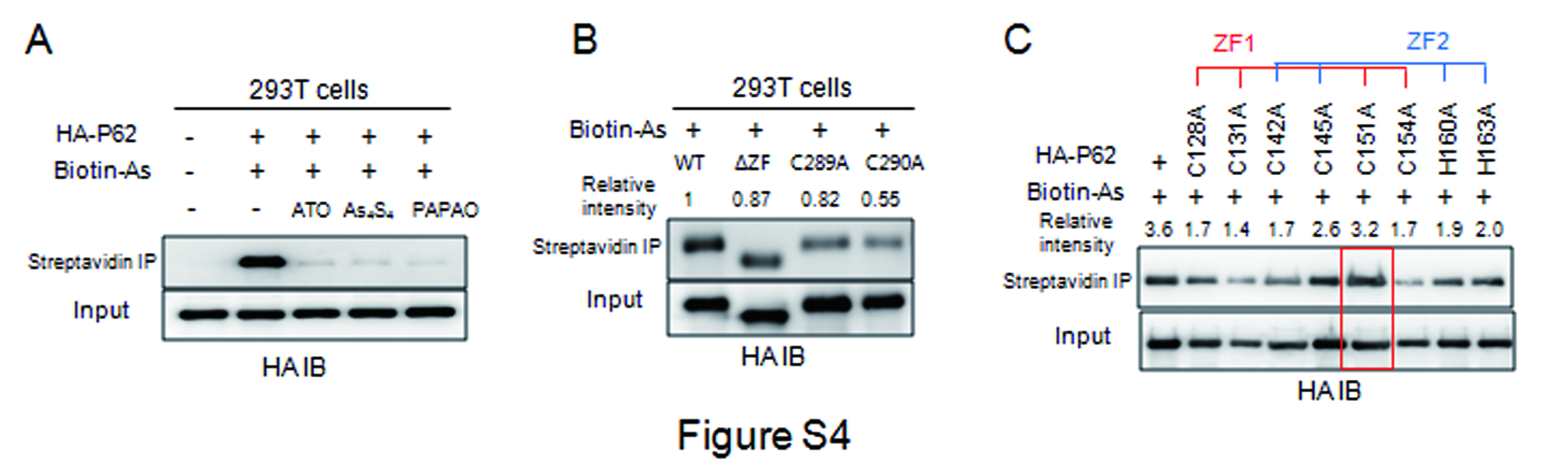

Supplement: Supplementary file 5 — Supplemental Figure 4 [file 41419_2018_998_MOESM5_ESM.tif]

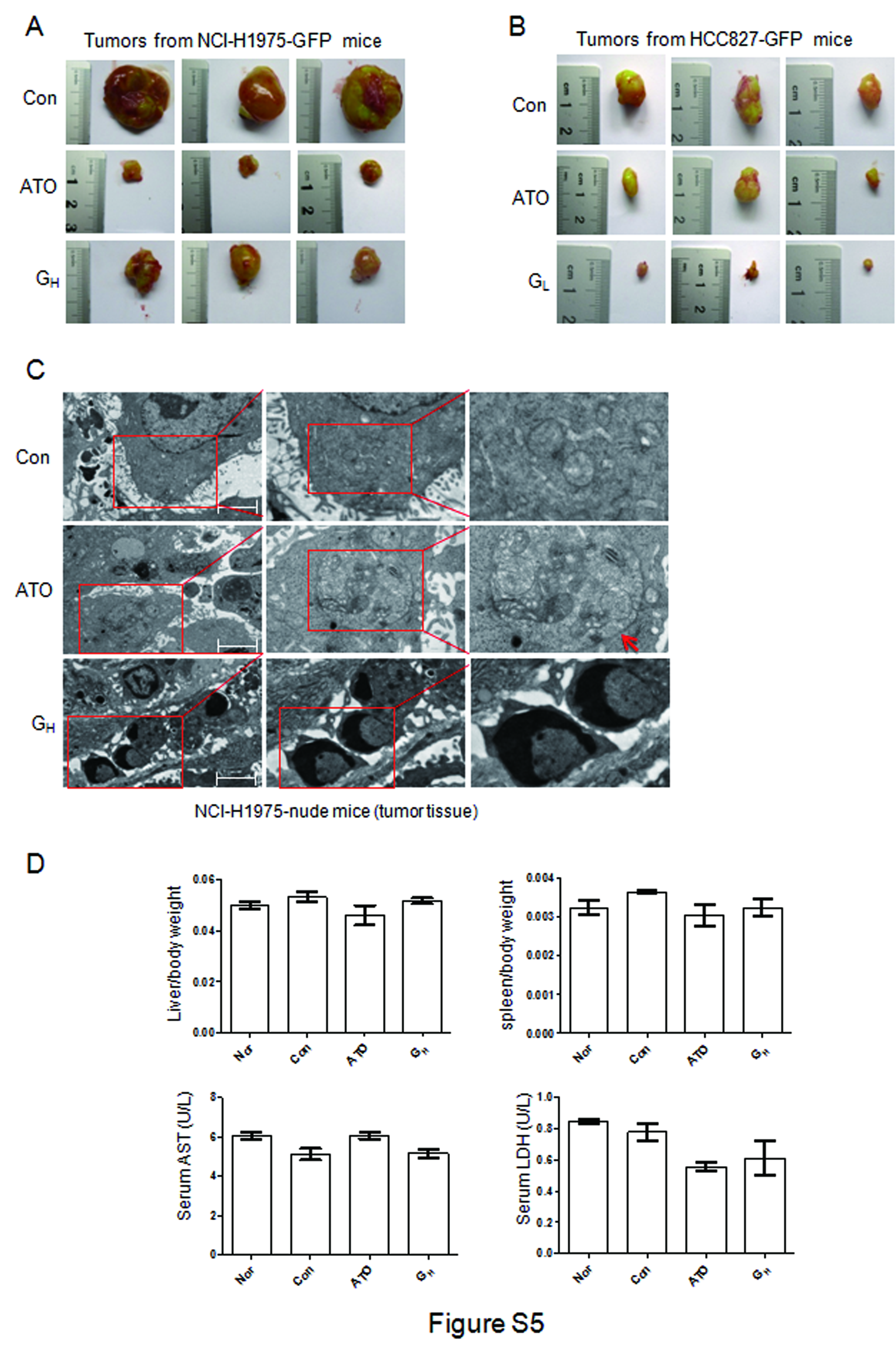

Supplement: Supplementary file 6 — Supplemental Figure 5 [file 41419_2018_998_MOESM6_ESM.tif]
